# Prayer, randomized controlled trials and healing: A response to Prof. Abraham Verghese

**DOI:** 10.4103/0019-5545.64589

**Published:** 2010

**Authors:** Chittaranjan Andrade, Rajiv Radhakrishnan

**Affiliations:** Department of Psychopharmacology, National Institute of Mental Health and Neurosciences, Bangalore - 560 029, India. E-mail: andradec@gmail.com; andrade@nimhans.kar.nic.in; 1 Department of Psychiatry, Yale University School of Medicine, New Haven, Connecticut, USA

Sir,

We are grateful to Prof. Abraham Verghese[1] for his interest in our critical examination of the scientific literature on blinded, randomized controlled trials on prayer and healing.[2] We wish to make the following observations in response to his letter:

1. We did not state that we "could not draw any conclusions" from our analysis. Rather, we argued with force and length that it is not possible to test the healing power of prayer through current methods of scientific enquiry.
2. The fact that the subject of our enquiry has been so extensively studied indicates that a large number of scientists across the globe did expect to uncover the "conclusive evidence" to which Prof. Verghese refers. As scientists, we sought to examine whether their efforts were justified. Our critical analysis provides the reasons for the opinion expressed by Prof. Verghese that their efforts were little more than "a wild goose chase".
3. We agree with Prof. Verghese that science and religion, like oil and water, do not mix; and that religious practice is associated with health benefits in diverse domains.[3] We indicated the latter in the preface to our paper[2] and with the very first reference that we had cited in it. However, as scientists, we are constrained to observe that the issues of cause, effect and confound remain to be resolved in this context.[4]
